# Spinach genomes reveal migration history and candidate genes for important crop traits

**DOI:** 10.1093/nargab/lqae034

**Published:** 2024-04-17

**Authors:** An Nguyen-Hoang, Felix L Sandell, Heinz Himmelbauer, Juliane C Dohm

**Affiliations:** Institute of Computational Biology, Department of Biotechnology, University of Natural Resources and Life Sciences, Vienna, Muthgasse 18, A-1190 Vienna, Austria; Institute of Computational Biology, Department of Biotechnology, University of Natural Resources and Life Sciences, Vienna, Muthgasse 18, A-1190 Vienna, Austria; Institute of Computational Biology, Department of Biotechnology, University of Natural Resources and Life Sciences, Vienna, Muthgasse 18, A-1190 Vienna, Austria; Institute of Computational Biology, Department of Biotechnology, University of Natural Resources and Life Sciences, Vienna, Muthgasse 18, A-1190 Vienna, Austria

## Abstract

Spinach (*Spinacia oleracea*) is an important leafy crop possessing notable economic value and health benefits. Current genomic resources include reference genomes and genome-wide association studies. However, the worldwide genetic relationships and the migration history of the crop remained uncertain, and genome-wide association studies have produced extensive gene lists related to agronomic traits. Here, we re-analysed the sequenced genomes of 305 cultivated and wild spinach accessions to unveil the phylogeny and history of cultivated spinach and to explore genetic variation in relation to phenotypes. In contrast to previous studies, we employed machine learning methods (based on Extreme Gradient Boosting, XGBoost) to detect variants that are collectively associated with agronomic traits. Variant-based cluster analyses revealed three primary spinach groups in the Middle East, Asia and Europe/US. Combining admixture analysis and allele-sharing statistics, migration routes of spinach from the Middle East to Europe and Asia are presented. Using XGBoost machine learning models we predict genomic variants influencing bolting time, flowering time, petiole color, and leaf surface texture and propose candidate genes for each trait. This study enhances our understanding of the history and phylogeny of domesticated spinach and provides valuable information on candidate genes for future genetic improvement of the crop.

## Introduction

Spinach (*Spinacia oleracea*, 2*n* = 2*x* = 12) is an esteemed leafy crop belonging to the Amaranthaceae family. Over the course of time from 1994 to 2021, its production has surged remarkably, multiplying approximately five-fold. Asian nations, particularly China, emerge as the predominant suppliers (1). Apart from its bountiful harvest, spinach has gained recognition for its high abundance of essential minerals, vitamins A, C and E, as well as flavonoids (2–5). Given the economic and nutritional value of this leafy green, there has been a pressing demand for clarifying its genetic characteristics (6). Consequently, important breakthroughs were achieved, starting with the first spinach genome assembly (genotype Viroflay) in 2014 (7), followed by the Sp75 assembly in 2017 (8). Both draft assemblies were based on Illumina short-read data. A combination of short-read and long-read data was used for generating improved assemblies published in 2021 (9,10). Subsequently, in 2021 and 2022, high-quality long-read reference genomes using PacBio and Hi-C sequencing data were generated (6,11). The previous comprehensive genome analyses, which incorporated re-sequencing data from hundreds of spinach accessions, were anticipated to untangle the intricate web of global population structure, migration routes, and the molecular mechanisms governing essential agronomic traits. However, despite the endeavors, a comprehensive understanding of these subjects remains elusive due to various limitations.

Concerning the phylogeny, genetic diversity and population structure of spinach, various studies employing simple sequence repeat (SSR) markers have categorized global spinach accessions into three primary groups, including cultivars from the Middle East, Europe or Asia. These groups often exhibited mixtures, particularly between Middle Eastern accessions and those from Asia or Europe (12,13). However, when studying large numbers of re-sequenced spinach genomes and analyzing a substantial number of single nucleotide polymorphisms (SNPs), a different picture emerged. Researchers such as Xu *et al.* have identified two main clusters globally for cultivated spinach. They designated ‘Group-2’ for Eastern Asian accessions and ‘Group-3’ for Central/Western Asian and European accessions (8). Similarly, Cai *et al.*, using a neighbor-joining dendrogram and a principal component analysis (PCA) plot based on over 500 000 SNPs, also found two distinct clusters. They denoted ‘Group I’ for spinach cultivars originating from Asian countries and ‘Group II’ for the ones in European countries and the USA (6). These SNP-based cluster analyses appear surprisingly less accurate compared to the SSR-based studies, considering the domestication of spinach occurred approximately 10 000 years ago (11). It is known that when crops are introduced to regions outside their centers of origin, new farming systems can result in selection pressures, creating bottlenecks and secondary diversity centers (14). Thus, with the extensive global breeding activities spanning thousands of years, it seems unlikely for spinach to exhibit only two distinct groups. To provide a definitive answer regarding the global population clustering of spinach accessions, further phylogenetic studies are neccessary, building upon the existing knowledge.

The migration history of spinach also remained an open question, yet to be further investigated through genome analyses. Prior to the release of high-quality reference genomes in 2021 and 2022, Ribera *et al.* shed light on spinach's migration paths based on historical records (15). According to the authors, spinach has reached Spain in the 11th century CE via Mediterranean countries in Africa, originating from Iran. However, there was no evidence of spinach migration from the Middle East to Eastern Europe. European spinach accessions were subsequently transported to North America after the 15th century CE. Additionally, the authors noted that spinach was brought to China in the 7th century CE, traversing through Afghanistan, India, and Nepal (15). It is worth mentioning that Ma *et al.* stands as the sole group of authors to present spinach migration history based on information derived from sequenced spinach genomes (11). Their findings confirmed the migration route from the Middle East to South and East Asia, while also indicating spinach's arrival in Eastern Europe, specifically North Macedonia, from the Middle East. However, due to the limited number of countries examined, the specific countries through which spinach migrated to reach North Macedonia remain unclear. Furthermore, the migration path from the Middle East to Western Europe via Northern Africa could not be definitively established (11). Consequently, a comprehensive analysis incorporating spinach accessions from a larger number of countries is necessary to further unravel the migration history of this crop.

Following the sequencing of the spinach genome, genome-wide association studies (GWAS) and RNA sequencing (RNA-seq) analyses were employed to pinpoint candidate genes responsible for governing crucial agronomic traits and other biological mechanisms (6,8,16–19). However, these analyses can yield extensive lists of genes associated with the traits, posing a challenge for the manual curation of gene functions. Consequently, an additional robust analysis that delves into whole genome sequencing becomes imperative to focus on smaller groups of candidate genes specifically linked to agronomic traits. This approach would greatly facilitate manual curation efforts and enable subsequent experimental validation to confirm gene functionality.

Here, we re-examine the genomes of 305 wild and cultivated spinach accessions as reported by Cai *et al.* (6). We use two independent phylogenetic analysis approaches to refine the comprehension of clustering patterns observed among global spinach accessions. By employing allele-sharing statistics and population tree estimation with admixture we shed light on previously undiscovered migration paths. Using the power of machine learning we introduce a method for identifying concise lists of candidate genes responsible for regulating important agronomic traits based on Extreme Gradient Boosting. Our results will be of importance for future breeding activities to improve the spinach crop.

## Materials and methods

### Sequencing data

The study utilized 305 sequencing data sets (fastq files) obtained from wild (*S. tetrandra* and *S. turkestanica*) and cultivated (*S. oleracea*) spinach, as generated by Cai *et al.* (6) (BioProject PRJNA598728). Accessions identifiers and country codes as used in our study are listed in Supplementary Table S1. For ease of reference, *S. tetrandra* and *S. turkestanica* accessions were abbreviated as STE and STU, respectively. The spinach reference assembly (GCA_020520425.1) utilized in this study was also sourced from Cai *et al.* (6). The fastq files were acquired from the Sequence Read Archive (SRA) database using the prefetch tool (v3.0.0) and subsequently processed with fastq-dump (v3.0.0), both of which are part of the SRA Toolkit collection (20).

### Read quality filtering and variant calling

To ensure data quality, Trimmomatic v0.40 was employed for read trimming from the fastq files (parameters: PE TruSeq3-PE.fa:2:30:10 LEADING:28 TRAILING:28 SLIDINGWINDOW:5:16 MINLEN:50) (21). The resulting paired and trimmed reads were then aligned to the reference genome using Bowtie2 v2.5.0, with the options -q –very-sensitive -S -X 1500 (22). Subsequently, the alignments were converted from SAM to BAM format by samtools v1.16.1 (using the command view -bS), and the resulting BAM files were sorted using samtools sort (23). These sorted BAM files served as inputs for the variant calling pipelines, which were based on gatk v4.3.0.0 (24), and were executed as follows.

The haplotypes were called using gatk HaplotypeCaller with the following options: -A BaseQuality, -A BaseQualityRankSumTest, -A FisherStrand, -A MappingQuality, -A MappingQualityRankSumTest, -A QualByDepth, -A RMSMappingQuality, -A ReadPosRankSumTest, -A ReadPosition, -A FragmentLength, -A StrandOddsRatio, and additional parameters (–min-pruning 1, –min-dangling-branch-length 1, –emit-ref-confidence GVCF). Individual gvcf files were then created and combined using gatk CombineGVCFs. Subsequently, the joint-genotyping process was performed using gatk GenotypeGVCFs with the combined gvcf files as input. Finally, the vcf file containing variants from the 305 accessions underwent quality filtering using gatk's hard filtering approach. The filtering criteria included the following expressions: ‘AC > 10’, ‘MQ > 30.0’, ‘DP > 1525’, ‘DP < 9150’, ‘SOR < 3.0’, ‘QD > 2.0’, ‘FS < 60.0’, ‘MQRankSum > −12.5’, ‘ReadPosRankSum > −8.0’, ‘BaseQRankSum > −12.5’, while also restricting alleles to be biallelic.

### Phylogenetic analysis

The phylogenetic trees were visualized using Newick utilities toolkit v1.6 (25). The tree construction based on k-mers employed Mash v2.0 (26) to generate subsequences of *k* = 22 from the quality-filtered forward Illumina reads of 256 cultivated accessions with country assignment (Supplementary Table S1) and of one wild accession (STU1) and to hash them into 100 000 min-hash sketches for pairwise genetic distance calculations. Initially, mash sketch was utilized to generate msh files from the trimmed fastq files (parameters -m 2 -r -s 100 000 -k 22), that were subsequently combined using mash paste with the -l option. The resulting combined msh file was used to calculate a pairwise distance matrix through the application of mash dist with the -t -l options. Finally, the phylogenetic tree was constructed using phylip v3.696 (D Fitch-Margoliash, J seed = 3, iterated 10 times) (27).

As a second approach we generated a phylogenetic tree based on variants. This Maximum Likelihood tree was constructed using 7023-bp pseudo-sequences derived from 404 086 unlinked variants of the same 257 accessions. To extract the unlinked variants (*r*^2^ ≤ 0.1), the linkage-disequilibrium-pruning (LD-pruning) option of plink v1.9 (28) was applied to the combined vcf file of the accessions obtained from the variant calling pipeline (–indep-pairwise 50 10 0.1). In this step, variants of *r*^2^ > 0.1 within a sliding window of 50 kb moved along the genome by 10 bp were pruned. Subsequently, pseudo-sequences were generated by concatenating the remaining variants using SNPhylo v20180901 (29), with the parameters -r -l 0.4 -M 0, saving them into a fasta file. The fasta sequences containing A, T, G, C for homozygous alleles and R, Y, M, K, S, W for heterozygous alleles (30) were used as input for a multiple sequence alignment using MAFFT v7.453 (31) with default settings. The consensus Maximum Likelihood phylogenetic tree was finally created using IQ-TREE v1.6.12 (parameters -m MFP -bb 1000 -bnni -st DNA). The random seed for the tree was 31081997 (32).

### Principal component analysis

To examine the clustering patterns of cultivated spinach accessions originating from various geographical regions, a parallel analysis was conducted alongside the construction of the phylogenetic trees. This analysis involved performing PCA on the unlinked variants derived from 256 cultivated accessions. The PCA was carried out using plink v1.9 (–pca). The resulting eigenvectors were plotted using ggplot2 (33).

### Admixture and migration analysis

Treemix v1.13 (34) and the ‘admixtools 2’ R package (35) were utilized to explore the admixture events and migration paths of the spinach accessions. In the initial step of the pipeline, positions with missing variants in the LD-pruned vcf file of 276 accessions (excluding 39 accessions with unknown origin) were removed using vcftools v0.1.16 (36) (–max-missing 1), generating 44 879 variants. Migration maps representing results from Treemix and F4 statistic calculations were constructed using the maps and ggplot2 R packages (33,37).

To identify the most likely scenario of migration between different geographic areas we conducted 98 replicates of the Treemix analysis with various k values of 1, 800, 1600, 2400, 3200, 4000 and 4800. The 98 trees had a varying number of migration edges ranging from 1 to 14. The ‘-bootstrap’ option was utilized in each run. The resulting output files from the 98 Treemix runs were analyzed to determine the optimal number of migration edges by employing the OptM() function from the OptM package (38). This allowed us to determine the migration value at which the likelihood score of the trees no longer significantly increased. Through this analysis, we estimated the optimal number of migration edges to be 1 (Supplementary Table S3, Supplementary Figure S3). The vcf file containing the unlinked variants with no missing alleles was converted into Treemix format using the vcf2treemix.sh script (39).

The formula for the F4 statistics was F4(A, B, C, D), where A represented the outgroup exhibiting high drift toward populations C and D as indicated by the Treemix analysis. Population B was identified as the source population proposed by Treemix, population C was a sister population within the same small clade as population D, and population D represented the target population for migration inferred by Treemix. Significant positive F4 values (*P*-values < 0.05) confirmed substantial gene flow (allele sharing) between populations B and D, while significant negative ones suggested gene flow between B and C. STE was chosen as outgroup (A) for all F4 calculations since it had the highest drift values to all other populations.

For F4 statistics calculations the bed, bim and fam files, which were generated from the LD-pruned vcf file with no missing data using plink (28) (–make-bed), served as input for the analysis. Initially, the f2 blocks were computed and loaded through the extract_f2() and f2_from_precomp(afprod = TRUE) functions. Subsequently, the f4() function was employed to identify gene flow between accessions from different countries.

### XGBoost models and variant selection

The variants in the vcf file of the 305 accessions, which were filtered by gatk and consisted of 4 580 829 biallelic SNPs and insertion/deletion polymorphisms (Indels), were recoded using plink (–recodeA). The resulting output was then merged with the phenotypic data sets obtained from Supplementary Data S6 of Cai *et al.* (6) to generate different combined data frames for the selected traits. A total of four traits were selected for the analysis, namely petiole color, leaf surface texture, bolting time, and flowering time. The bolting and flowering time traits were categorized into two classes with similar sample sizes.

The processes of building and evaluating XGBoost models and of identifying the most significant variants were described accompanied by the corresponding Python code (see Data Availability). In summary, the models were constructed using the XGBClassifier() class from the xgboost module (40), utilizing different training data sets that accounted for 80% of the combined data (with both phenotypic and genotypic values) of the traits. The train_test_split() function from the sklearn module was employed for data splitting (41). To optimize the models, parameters such as ‘learning_rate,’ ‘max_depth,’ ‘min_child_weight,’ ‘gamma’, ‘subsample’, and ‘colsample_bytree’ were fine-tuned using the fmin(algo = tpe.suggest, max_evals = 100) function from the hyperopt module (42). The resulting optimized models were saved using the joblib.dump() function (43).

To assess the models' accuracy with varying training data, the combined data frame comprising genotypic and phenotypic values for each trait was split into training and testing data in an 8:2 ratio using 100 different random seeds. The optimized models were then fitted to the various training data sets and predictions were made on the corresponding testing data. The accuracy of the models was calculated using the accuracy_score() function from the sklearn module (41). By repeating this fitting and prediction process, all of the resulting accuracy scores were obtained for each optimized model. These scores were then used to visualize the accuracy ranges and calculate the mean accuracy of the models.

To determine if the optimized models outperformed their corresponding baseline models (constructed using the class DummyClassifier(strategy=‘most_frequent’) from sklearn), the 5 × 2 cv paired *t*-test from the mlxtend module was utilized (44,45). Models with *P*-values < 0.05 were considered superior to their baselines.

For the identification of the most important variants associated with the traits, after each fit, the ‘model_name.get_booster().get_score(importance_type=’gain‘)’ command was used to extract a list, containing the variants with scores (considered as ‘appeared’ in predictions and increasing the prediction accuracy) and without scores (NA) (marked as ‘not appeared’). Finally, the 100 variant lists, containing variants with and without scores, were merged into a data frame, and the frequency of variant usage was determined. Variants that ‘appeared’ > 20 times (this threshold was arbitrarily chosen) in the predictions were selected for mapping using chromoMap v0.4.1 (46) and further analyses.

### Variant annotation and candidate genes

To annotate the variants with the highest impact and identify those occurring within genes, SnpEff v4.3t (47) was used. Initially, a spinach database was constructed using the Monoe-Viroflay reference genome, gene, CDS and protein annotation files obtained from the website spinachbase.org (snpEff build). Subsequently, the vcf file containing the crucial variants was annotated using the ‘snpEff ann’ command. For functional analysis, the amino acid sequences associated with the genes encompassing the variants were utilized as queries in command-line-based BLASTp (Uniprot database) (48) with default parameters. Amino acid sequences lacking a name or significant BLAST hit were excluded from further examination.

To visually represent the genotype distribution of variant positions considered pivotal for predictions based on XGBoost across diverse phenotypic groups (Supplementary Table S5), we conducted genotype recoding of these variant positions via the utilization of plink v1.9 (28). We assigned values of 0 for homozygous reference genotype, 1 for heterozygous genotype, 2 for homozygous alternative genotype and −1 for missing genotype. Subsequently, we harnessed the Heatmap() function integrated within the ComplexHeatmap R package to perform hierarchical clustering of both the variants and the spinach accessions (49).

Additional variants correlated with the crucial variants were found via the computation of Pearson's correlation coefficients between each pivotal variant and the entire variant set encompassing 4 580 829 variants. This was achieved using the ‘corrwith(method = ‘pearson’)’ function in Python 3.10. Variants exhibiting a correlation value < 97% were omitted from consideration. Correlated variants were annotated, and functions of the genes associated with them were examined using the same process as above.

### Computing resources

The analyses were done using computing nodes at the Vienna Scientific Cluster (operating system: AlmaLinux) managed by the Slurm job scheduling system, each equipped with a 64-core CPU having base frequency of 2.0 GHz, memory from 512 GB to 2 TB, and a 2-TB NVME disk. Additionally, a high-performance Linux computing cluster under CentOS 6.7 and CentOS 7 equipped with nine computing nodes (having up to 56 cores and 1 TB of RAM) was used.

### Coding/scripting/editing

The R commands and Python codes were executed in RStudio v2023.03.0 9 (R 4.2.2) and Jupyter Notebook v6.5.3 (python 3.10). For drafting the manuscript language editing was done using ChatGPT v3.5 (OpenAI). Final manuscript writing was done without artificial intelligence.

## Results

### Spinach phylogeny and population structure

We applied two phylogenetic approaches on a subset of 256 cultivated spinach accessions for which geographic information was available and one *S. turkestanica* wild accession (STU1) from Cai *et al.* (6) (Supplementary Table S1). In the first approach, the Fitch-Margoliash algorithm was applied on pairwise distances of collections of representative k-mers extracted from the sequencing data of these accessions. The second approach utilized genomic variants after mapping the sequencing data to the spinach reference genome (the inbred line Monoe-Viroflay (6)) and a Maximum Likelihood consensus tree calculation (see Methods).

**Figure 1.**
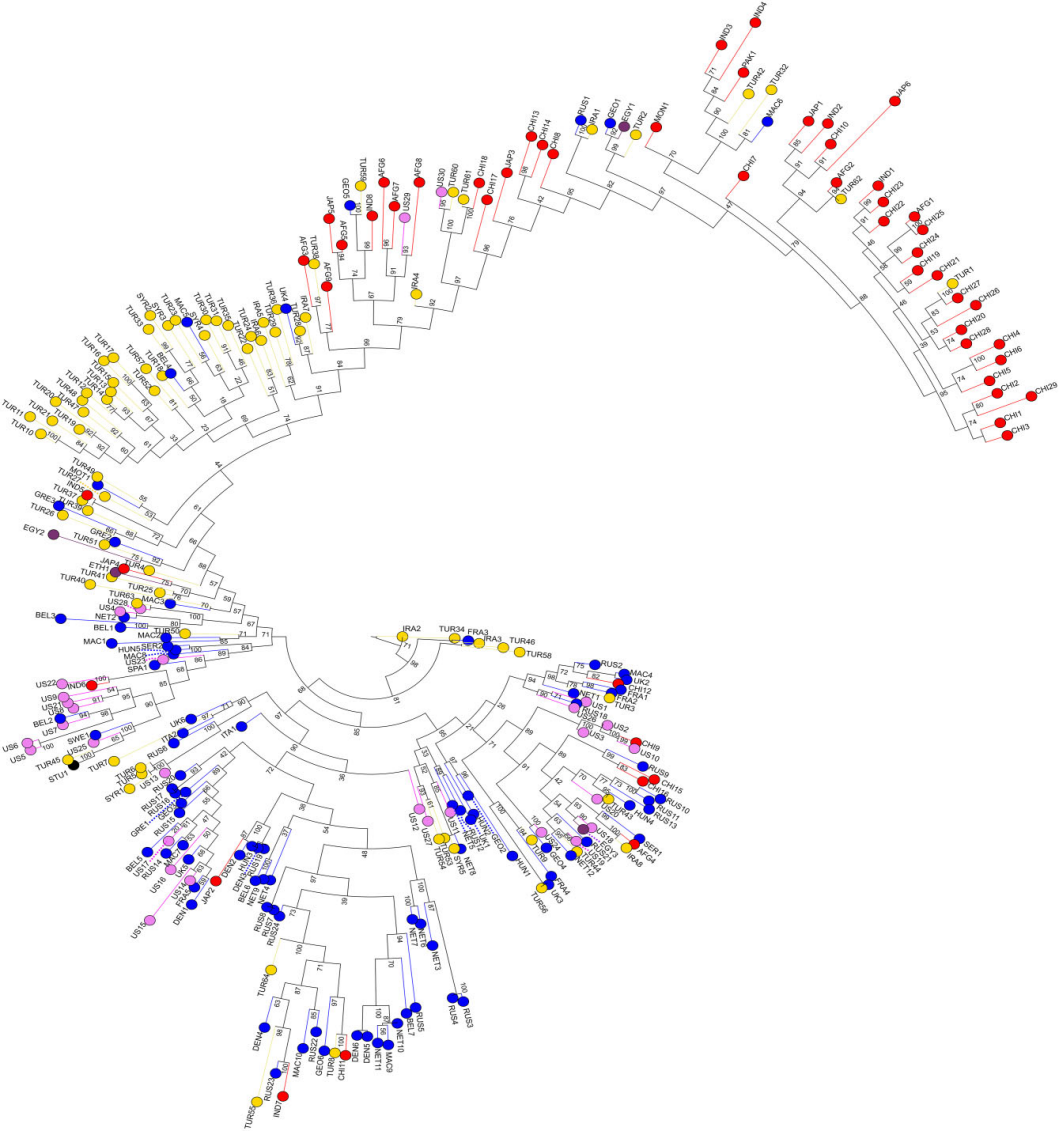
Maximum likelihood variant-based consensus tree rooted at IRA2 showing 256 cultivated spinach accessions from Asia (red), Europe (blue), the Middle East (yellow), the US (pink), Africa (purple) and one wild accession (STU1).

The variant-based tree (Figure 1) showed three primary groups encompassing accessions from Asia, Middle East (here defined as Iran, Syria and Turkey), and a mixture of European/US accessions. The Asian subtree was most clearly separated from the rest of the tree. Next to the Asian subtree, at the transition to the Middle Eastern subtree, there were several accessions from Afghanistan and India. The main features of the variant-based tree were recapitulated in the k-mer-based tree (Supplementary Figure S1), i.e. Asian accessions clustering together, likewise grouping and mixture of European and US accessions, and most of Middle Eastern accessions appearing close to each other.

The phylogenetic trees also provided insights into potential migration events, as evidenced by accessions positioned outside their geographical clusters, e.g. Afghan accessions at the root of the Asian subtree next to the Middle Eastern cluster and Turkish accessions in the Asian cluster. These mixtures suggested potential migration routes from the Middle East towards Eastern/Southern Asia via Afghanistan. Regarding migration to Europe, accessions from Greece, North Macedonia and Montenegro appearing in proximity to the Middle Eastern cluster may support a migration path from the Middle East to Europe via these countries.

**Figure 2.**
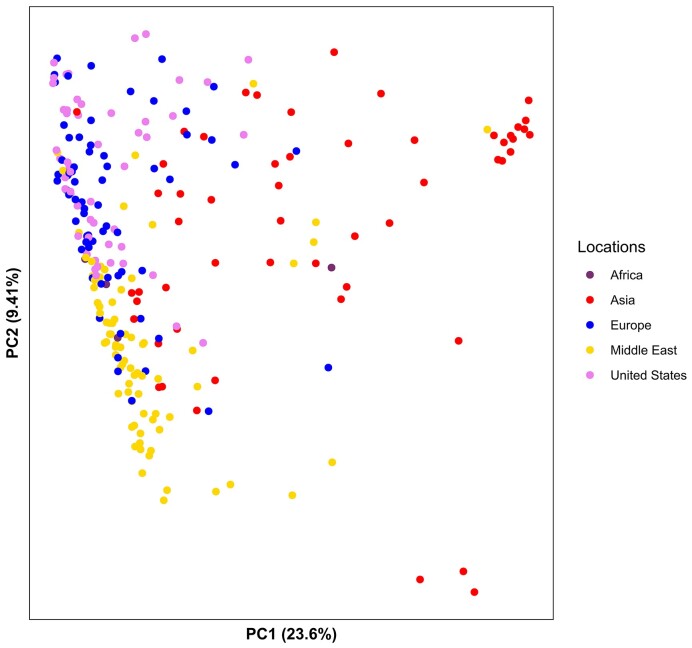
Principal component analysis based on genomic variation in cultivated spinach accessions.

Clustering patterns supporting the phylogenetic trees were also observed in a principal component analysis (PCA) based on 404 086 unlinked variants (Figure 2, Supplementary Table S2). Middle Eastern (yellow), European (blue) and US (pink) spinach accessions coalesced to form a large cluster separated from most of the Asian (red) accessions. Middle Eastern accessions predominantly occupied one-half of this cluster, while European and US accessions were heavily mixed among each other in the other half. Closer inspection revealed that all the red data points appearing close to the Middle Eastern cluster were from Afghanistan, a country adjacent to the Middle East, or from India (Supplementary Figure S2).

### Spinach migration history

To get further insights into the migration history of spinach we investigated admixture events and directional gene flows among wild and cultivated spinach accessions based on the allele frequencies of 44 879 unlinked variants (with no missing allele). We employed a heuristic maximum likelihood search as implemented in Treemix enabling inference of the genetic relationships among spinach accessions worldwide. In this type of tree visualization, branches represent populations, and links between branches (‘migration edges’) represent admixture events between populations unable to be explained by the tree model (50). We performed 98 replicated tree calculations using varying numbers of migration edges (Supplementary Table S3, Supplementary Figure S3).

**Figure 3.**
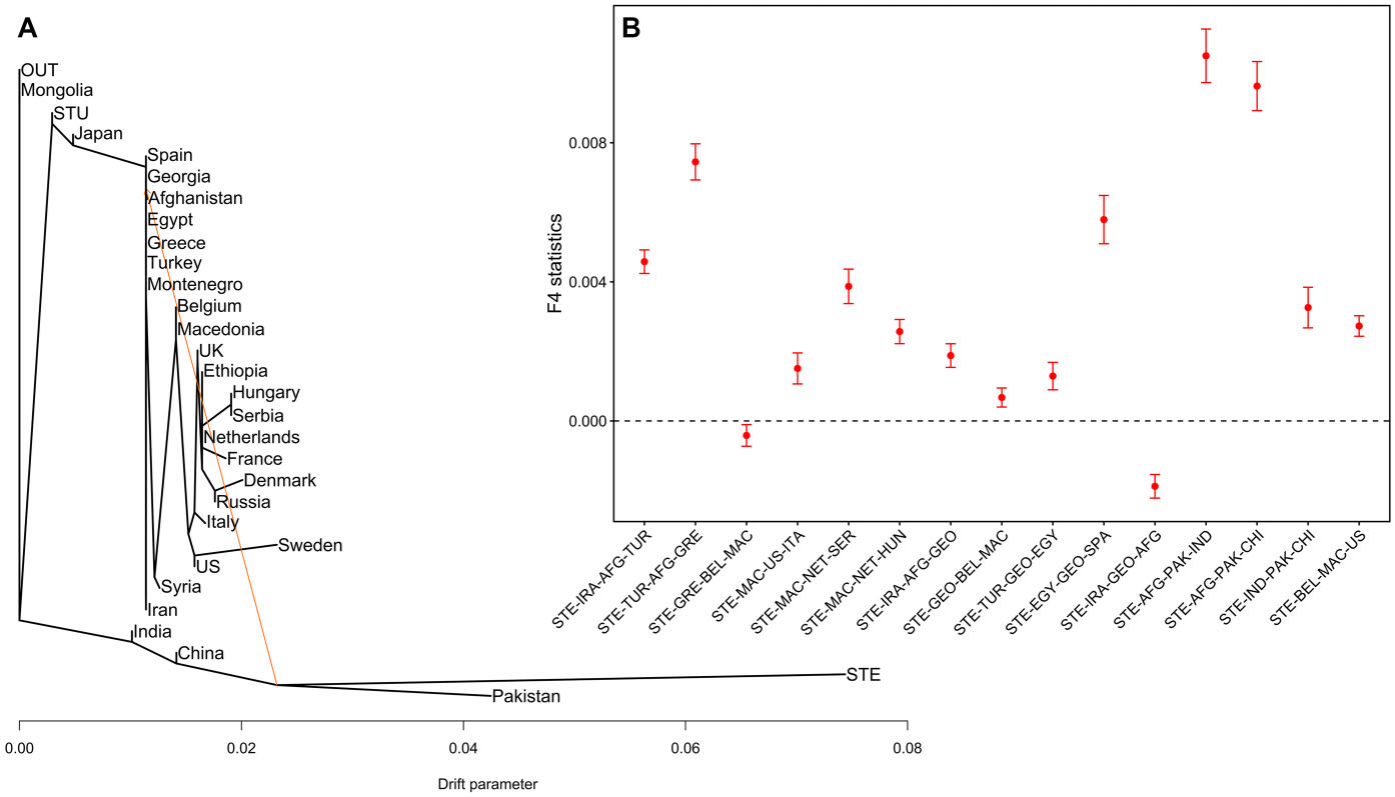
Analysis for potential gene flow among spinach populations in different countries. (**A**) The population tree with admixture constructed using Treemix shows genetic relationships among the populations and a possible migration edge depicted as arrow. The outgroup (OUT) was created by two STU accessions. (**B**) The data points are the average F4 statistics across all variants and the error bars are standard errors, STE was used as outgroup.

The clustering patterns and drift values in the optimal population tree (Figure 3A), were used for the computation of F4 allele-sharing statistics to address potential biases that could arise from Treemix analysis alone (50) (Supplementary Table S4). As notation for these statistics we used F4(A, B, C, D), where B represented the source population and D the target population for migration as inferred by Treemix. Significant positive F4 values imply substantial gene flow (allele sharing) between populations B and D which we here refer to as F4 values of ‘B−D’ using country abbreviations.

The admixed tree topology, with a highly weighted migration edge and the statistical significance of positive F4 values (Figure 3) provided evidence of spinach's migration routes.

**Figure 4.**
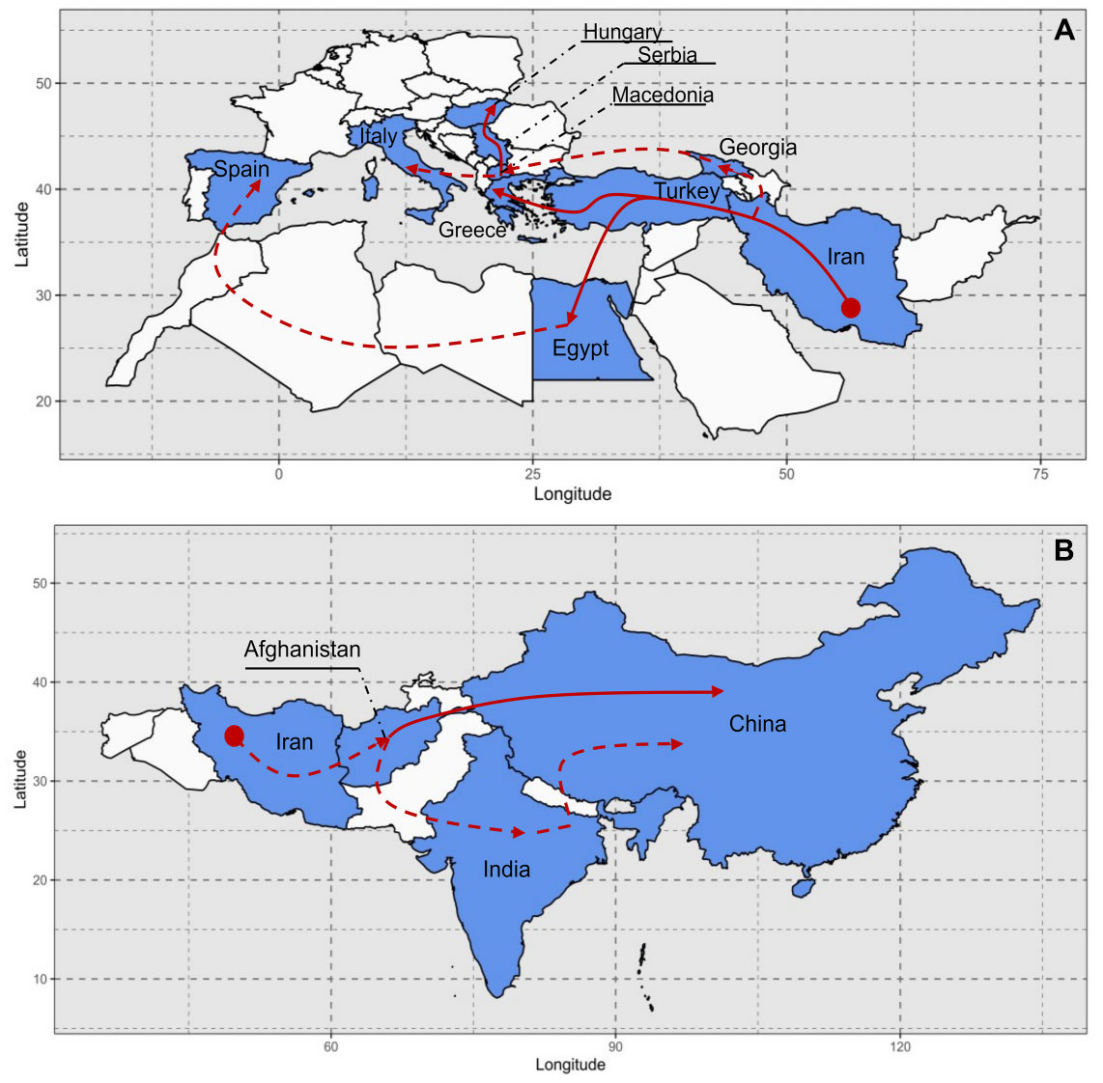
Reconstruction of spinach migrations from the Middle East to Europe (**A**) and Asia (**B**). The maps were drawn using the results from admixed population tree building and F4 statistic calculations. The dashed paths had lower confidence than the solid ones due to lack of sequencing data from interjacent countries or lack of support from F4 statistics.

Both the population tree and the F4 statistics supported a close genetic relationship of spinach accessions from Iran, Turkey, and Greece with F4 for IRA-TUR and TUR-GRE showing significant positive values. This led us to postulate that spinach started its journey from Iran, passing through Turkey, with Greece acting as a gateway to Eastern Europe (Figure 4A). Although Greece and North Macedonia are neighboring countries and genetic exchange may be likely, we observed no significant F4 values for GRE-MAC. Nevertheless, migration routes of spinach from North Macedonia to other European countries encompassing Serbia, Hungary, and Italy were supported by significant F4 values and the tree topology.

The population tree also suggested that after its arrival in Belgium, spinach found its way across the Atlantic, establishing a new home in the United States. The significant positive F4 value for BEL-US supported this migration path. Noticeably, our analysis revealed another migration path to Eastern Europe. Specifically, Eastern Europe may have received spinach via a route originating from the Middle East (Iran) and passing through Georgia before reaching North Macedonia indicated by significant positive F4 values for IRA-GEO and GEO-MAC. Along this line, the population tree showed the Macedonian population as the descendant not only of the Greek and Turkish ones but also of the Georgian one.

The data also suggested a migration path from Turkey to Western Europe, specifically Spain, with African countries, including Egypt, playing a role in facilitating this movement. Although we lacked sequenced accessions from other Mediterranean countries in Africa, the F4 statistics provided evidence of gene flow between Turkey-Egypt and Egypt-Spain, supporting this route (Figure 4A).

Regarding spinach's migration from the Middle East to Asia, Afghanistan emerged as a crucial point, through which the plant may have ventured from the Middle East to Southern and Eastern Asia (Figure 4B). India may have served as spinach's intermediate destination before reaching China supported by significant positive F4 values for AFG-IND, AFG-CHI and IND-CHI as well as by a highly weighted migration edge between AFG and the Indian/Pakistani/Chinese clade in the population tree.

The tree topology shows Iranian spinach as ancestral population for populations in various geographic directions, i.e. Turkey, Georgia, Afghanistan and Egypt, from where the crop spread to more distant regions suggesting Iran as the likely center of spinach domestication.

### XGBoost model building for spinach phenotype predictions

While previous studies used classical GWAS or gene expression analyses for associating genotypes to phenotypes resulting in extensive gene lists (6,8,16–19) we set out to analyze the sequenced genomes and their phenotypes by building predictive machine learning models based on genomic variation using Extreme Gradient Boosting (XGBoost). Two quantitative traits (bolting time, flowering time) and two qualitative traits (petiole color, leaf surface texture) were selected for model building. The samples studied by Cai *et al.* (6) had been obtained from a single location and growing season. Thus, the phenotypic data collections for bolting and flowering time could not guarantee the reduction of environmental noise, as quantitative traits are widely recognized to be influenced by environmental factors. Consequently, we simplified the continuous phenotypic values by dividing them into classes and chose accessions on both sides of the phenotype distribution to ensure that noise in the data set would not affect the predictions. XGBoost can theoretically be used as a regression model, i.e. working directly with quantitative data. This is a more difficult task than a classification problem meaning that a very large and balanced phenotypic dataset with a low error margin would be necessary to achieve trustworthy results. The following two classes were assigned to the traits: Class 0 represented individuals with green petioles, rough leaf surface, early bolting (40–120 days), or early flowering (93–146 days), respectively, while class 1 encompassed plants with purple petioles, smooth leaf surface, late bolting (156–203 days), or late flowering (177–230 days), respectively (Table 1). Genotypic and phenotypic data were combined for each of the four traits.

**Table 1.** Phenotypic classes and ranges for the four selected qualitative and quantitative traits

Agronomic trait	Class/range
Petiole color	Green(0), Purple (1)
Leaf surface texture	Wrinkled (0), Smooth (1)
Bolting time	40–120 days (0), 156–203 days (1)
Flowering time	93–146 days (0), 177–230 days (1)

**Figure 5.**
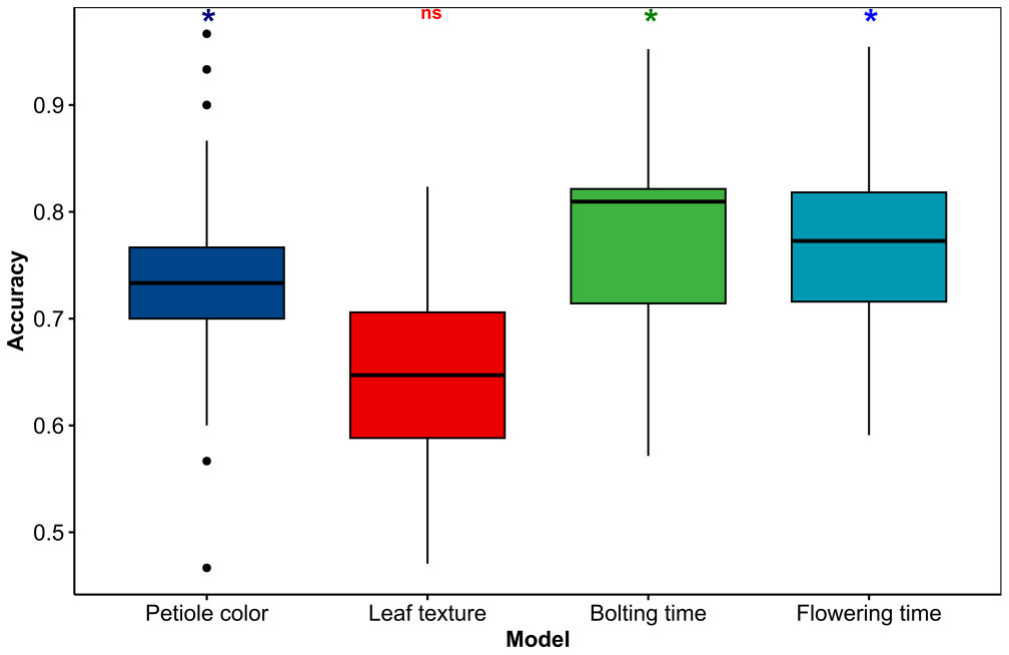
The XGBoost models built for the four traits exhibited accuracy ranges of > 52%, which represented the highest accuracy score among the four baseline models. The baseline models predicted only one class and were used as baselines for evaluating the XGBoost models. The asterisks above each box plot show significant improvements of the XGBoost models compared to their baselines by the paired 5 × 2 cv *t*-test (*P* < 0.05). The model performance for leaf surface texture prediction was not significantly higher than the baseline model (ns).

We generated and evaluated XGBoost models after dividing the data into training sets and test sets for each trait. When different training data sets are supplied to the XGBoost models, the prediction accuracy and the resulting most important variants for phenotypic class predictions can change. Thus, for each trait we extracted 100 distinct training sets each comprising 80% of the total data per trait. Subsequently, we fitted models to all 100 training sets generating ranges of accuracy scores. The models achieved mean prediction accuracies of 73.5%, 65.4%, 77.5% and 76.8% for predicting petiole color, leaf surface texture, bolting time, and flowering time, respectively, outperforming the baseline accuracy (Figure 5).

However, the 5 × 2 cv paired *t*-test revealed that only the models for predicting petiole color, flowering time, and bolting time were statistically significant improvements over their respective baseline models, as indicated by *P*-values < 0.05 (Table 2).

**Table 2.** Evaluation of models’ performance based on the 5 × 2 cv paired *t*-test. The asterisk (*) indicates a significance level of *P* < 0.05

Trait	*t* statistics	*P* values
Bolting time	3.427	0.019*
Flowering time	2.851	0.036*
Petiole color	3.873	0.012*
Leaf surface texture	0.689	0.521

### Identification of genes regulating spinach agronomic traits

From the 100 independent XGBoost models for each trait we collected the variants that resulted in a gain in prediction accuracy in at least 21 models and hypothesized that they are likely to possess biological functions that regulate the traits.

In total, we gathered 93 variants that were considered vital for predicting agronomic traits under study, namely 16, 17, 33, and 27 variants for bolting time, flowering time, petiole color, and leaf surface texture, respectively. Since only unlinked variants were considered up to this point, we calculated Pearson's correlation coefficients between each of these 93 variants and the complete variant set. We found 11, 14, 32 and 3 additional variants that were highly correlated (> 0.97) with the 93 variants deemed crucial for regulating bolting time, flowering time, petiole color, and leaf surface texture, respectively (Supplementary Table S5).

**Figure 6.**
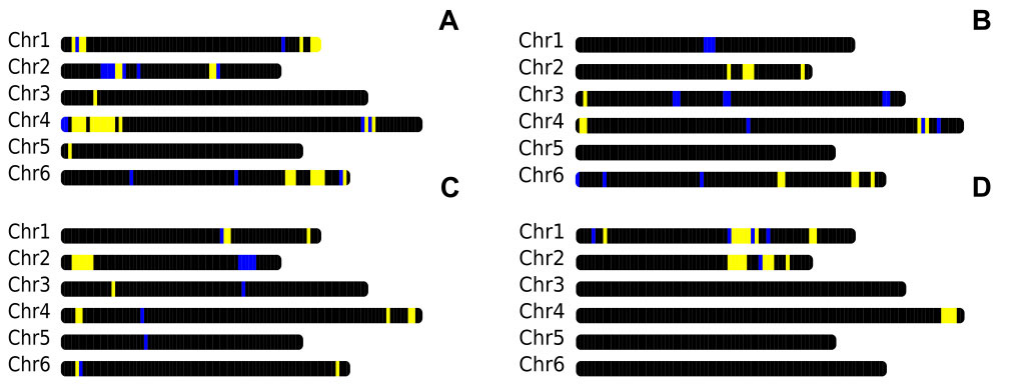
Chromosomal locations of the most important variants for agronomic trait predictions including petiole color (**A**), leaf surface texture (**B**), bolting time (**C**) and flowering time (**D**). Yellow blocks represent gene-related variants, while blue blocks show intergenic variants. The width of blocks is not to scale but illustrates the number of variants. In the petiole color trait, the right tip of Chr1 has eight variants, and the left tip of Chr4 has six variants.

Comparing the variant positions to the positions of the gene annotations, we identified a set of 42, 15, 17 and 24 variants associated with genes responsible for petiole color, leaf surface texture, bolting time and flowering time, respectively (including correlated variants). Most of these variants (82 of 98) were discovered in the upstream, downstream, and intronic regions of the genes (Supplementary Table S5). We mapped these 98 gene-associated variants onto the chromosomes of spinach to display their genomic distribution (Figure 6). For petiole color, chromosomes 1, 4 and 6 showed most of the variants, for leaf surface texture chromosomes 2, 4 and 6, and for bolting time chromosomes 2 and 4. Intriguingly, chromosome 1, identified as spinach's sex chromosome by Ma *et al.* (11), emerged as a primary regulator for flowering time (containing 17 variants) followed by chromosome 2 (9 variants). Chromosome 5 exhibited minimal impact on all traits, with zero or one variant.

Conducting functional examinations on genes associated with the detected variants unveiled a set of genes that were previously unknown in their potential regulatory capacity for spinach agronomic traits (Table 3). The gene suggesting crucial roles in the control of bolting time encoded the auxin-binding protein 19a (ABP-19a). The genes suggested as influential for spinach flowering time included *ABP19A*, *FLZ3* and *ESD4*. Genes that might govern spinach petiole color were intricately involved in the biosynthesis and accumulation of anthocyanin. Additionally, we identified genes that might influence leaf surface texture by regulating cell division, auxin signaling, and interaction with the cytoskeleton (see Discussion).

**Table 3.** List of candidate genes carrying the important variants to trait regulation

Trait	Gene	Location	Position
Bolting time	Auxin-binding protein (*ABP19A*)	Upstream	Chr1_Pos86175101
Flowering time	Auxin-binding protein (*ABP19A*)	Upstream	Chr1_Pos86177123
	FCS-like zinc finger 3 (*FLZ3*)	Downstream	Chr2_Pos91054045
	Ubiquitin-like-specific protease ESD4 (*ESD4*)	Upstream	Chr1_Pos86611549
Petiole color	Sm-like protein LSM1B (*LSM1B*)	5′ UTR	Chr1_Pos131172658
	Axial regulator YABBY 5 (*YAB5*)	Upstream	Chr2_Pos80586117
	Small heat shock protein (*HSP22*)	Intron	Chr4_Pos9258969
	Probable protein phosphatase 2C 5 (*PP2C05*)	Intron	Chr4_Pos17151789
	NAC domain-containing protein 71 (*NAC071*)	Downstream	Chr6_Pos148214276
	Serine decarboxylase 2	Downstream	Chr4_Pos160554005
Leaf surface texture	Triphosphate tunnel metalloenzyme 3 (*TTM3*)	Upstream	Chr2_Pos83772929
	Protein PHOX1 (*PHOX1*)	Downstream	Chr2_Pos83864920
	G2/mitotic-specific cyclin-1	Downstream	Chr4_Pos168318794
	Bifunctional monothiol glutaredoxin-S16 (*GRXS16*)	Downstream	Chr6_Pos137481996
	Cytochrome P450 (*CYP78A3*)	Downstream	Chr6_Pos145342003

**Figure 7.**
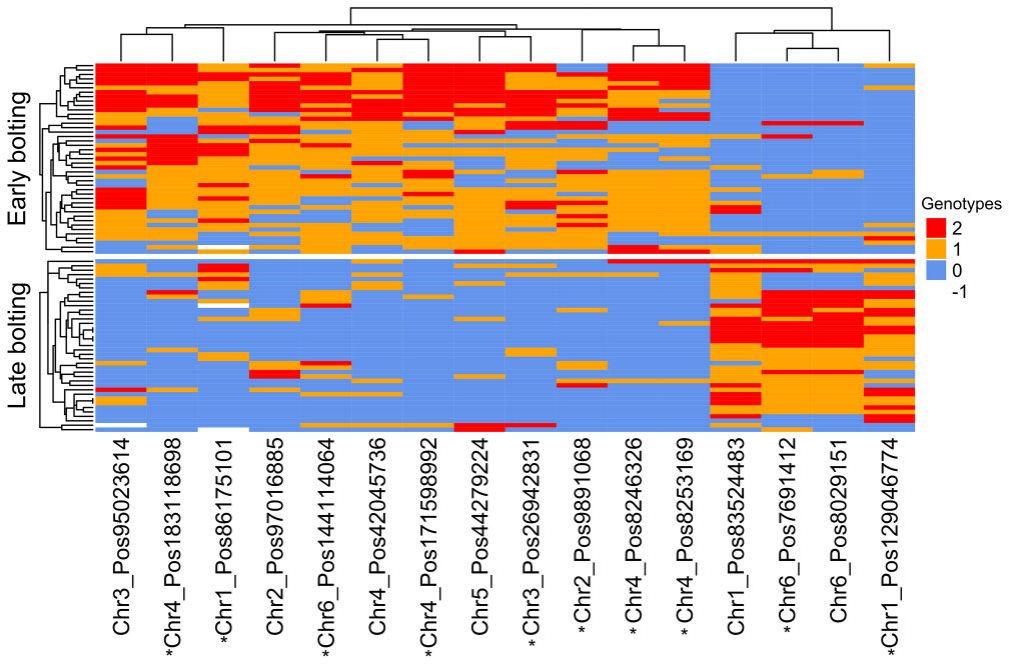
Genotype distribution patterns among phenotypic groups associated with bolting time accomplished through the clustering of accessions and variant positions by genotypes. The genotypes were recoded as ‘0’ for homozygous reference, ‘1’ for heterozygous, ‘2’ for homozygous alternative, and ‘−1’ for missing genotype. The horizontal axis represents variant positions and the vertical axis shows the individuals in each phenotypic group. Variants associated with genes are highlighted with asterisks.

In addition to the functional assessment of the potential candidate genes, we analyzed the genotype distributions of the variant positions that were identified as crucial for XGBoost-based predictions across various phenotypic groups. Instead of single variants, it was the collective of variants that contributed to distinctive genotype distribution patterns. Focusing on variants that were found in the vicinity of genes, disparities in genotype distributions among phenotypic groups were apparent for all four traits: one phenotypic group predominantly exhibited homozygous and heterozygous variant genotypes, while the other group predominantly showed homozygous reference genotypes. The separation was especially obvious for bolting time (Figure 7), flowering time (Supplementary Figure S4), and petiole color (Supplementary Figure S5), and less pronounced for leaf surface texture (Supplementary Figure S6).

## Discussion

The present study has made relevant contributions to the interpretation of spinach phylogeny, the understanding of its migration history, and the discovery of candidate genes for future spinach breeding projects.

In terms of spinach phylogeny, our research supports and expands upon previous SSR-based findings regarding spinach global clustering patterns, using two algorithms for phylogenetic tree construction and a high number of 404 086 variants for PCA analysis. Specifically, our results align with the observations made by Kuwahara *et al.* and Bhattarai *et al.*, who identified three main spinach clusters worldwide: East Asia, West Asia (including the Middle Eastern countries), and Europe. These studies also noted the mixtures of West Asian and European accessions and US and European accessions (12,13). Consistently, our phylogenetic analysis and PCA results support the presence of these three primary groups in spinach (Asia, Middle East and Europe/US), as well as genetic similarity between European and Middle Eastern accessions, along with the mixture of European and US spinach (Figures 1, 2).

Furthermore, our phylogenetic analysis and PCA revealed spinach clusters that offer an explanation for the findings of previous studies, which were based on a larger number of SNPs but exhibited limited spinach clusters worldwide or different genetic relationships among global spinach populations compared to our study. For instance, Shi *et al.* identified separate clusters for European, Asian, US, and Turkish accessions but proposed a closer genetic affinity between European and ‘Asian’ accessions, where the term ‘Asian’ also included Middle Eastern accessions from Iran, Iraq, and Syria. The similarity between Middle Eastern and European accessions likely influenced their clustering results (51). Similarly, Cai *et al.* observed close genetic relationships between Middle Eastern accessions (from Turkey, Iran, and Syria) and European/US accessions, resulting in the clustering of all these accessions within ‘Group II’. Consequently, their phylogenetic tree consisted of only two groups (Asia and Europe-North America), limiting the insights into spinach history. Among studies using SNPs for phylogenetic analysis, Xu *et al.* achieved relatively consistent clustering results that aligned with both SSR-based studies and our own findings. They grouped accessions from Central and Western Asia near European accessions. However, the absence of tree membership information for Middle Eastern accessions hindered a comprehensive understanding of the grouping patterns (8).

In general, assigning cultivated spinach to groups of geographical origin remains somewhat arbitrary due to the continuous and adaptive nature of domestication, along with exchange of material during breeding activities across different locations. However, based on our results the postulation of three primary groups for spinach seems to reflect a reasonable approximation of the complex distribution of the crop.

Our phylogenetic study also highlights instances where spinach accessions cluster outside their expected geographic groups, offering hints regarding spinach's migration history that were supported by our admixed population phylogenetic analysis and allele-sharing statistical analyses.

The present study complements archaeological evidence and the research conducted by Ma *et al.*, providing specific insights into the routes through which spinach traveled from the Middle East to Europe (11,15,52). Specifically, Ma *et al.* broadly indicated that spinach was introduced to Europe via North Macedonia. Our findings utilizing the F4 statistics and the admixture population tree support this notion and furthermore suggest the possibility of spinach being brought to North Macedonia from Georgia (Figure 3). From North Macedonia, spinach then spread to other European countries. Our study also aligns with Ma *et al.*’s assertion that Belgium served as the source of spinach introduction to the United States (11). Although our study lacked spinach accessions from Libya, Algeria, Tunisia and Morocco, we provided genome-based evidence supporting the migration path from Northern Africa to Western Europe, as documented in historical records (15,52) by detecting gene flow between Egypt and Spain. Regarding spinach migration from the Middle East to Asia, our findings align with Ma *et al.*’s research, indicating that spinach reached China through India, with a potential route through Nepal as suggested by Ribera *et al.* Furthermore, we found hints for a second migration route to East Asia, where spinach traveled directly from Afghanistan (in Central Asia) to China (Figure 4).

The variants from the genomes of spinach accessions in our study not only enhance our understanding of spinach phylogeny and history but also provide valuable information for future genetic development by presenting lists of candidate genes newly identified to regulate spinach agronomic traits. However, although there are published studies supporting the candidate genes (see below), future experimental validations for the effects of the genes on their proposed respective traits must be conducted.

Given the expectation that intricate phenotypic traits in agriculturally important crop plants are governed by a diverse spectrum of genomic variation, we employed machine learning models rather than conventional GWAS to decipher genotype-phenotype interactions. Our methodology successfully predicted four phenotypes exclusively based on their distinctive variation patterns. Groups of variants per phenotypic class could be even visually distinguished in genotype heatmaps for each trait. Notably, none of these variants were discovered in a previous study using GWAS (6). The outcomes of our analyses indicate that these traits are probably governed by a complex interplay of factors within the spinach genome. Subsequently, we extracted the variants that led to a gain in prediction accuracy, aiming to identify the associated genes.

In the context of genes associated with the control of bolting time, our study has uncovered one intriguing candidate gene encoding ABP19a. Auxin-binding proteins (ABP) play a crucial role in modulating polar auxin transport by inhibiting the internalization of PIN proteins (53). Within our study, we identified the gene coding for the protein ABP19a, which has been reported to exhibit differential expression during floral induction and flower bud growth in various plant species, including *Pyrus pyrifolia* and *Vaccinium corymbosum* (54,55). Noticeably, ABP1, a germin protein similar to ABP19a, has been implicated in bolting time control in *Arabidopsis thaliana* (56). Therefore, the ABP19a-encoding gene emerges as a relevant candidate worthy of experimental investigation, as it potentially contributes to the regulation of bolting in spinach.

The present study has also identified candidate genes encoding proteins that might be crucial for controlling flowering time, with connections to auxin transport, a zinc finger protein, as well as involvement in essentials functions of the SUMO (small ubiquitin-like modifier) pathway. Specifically, ABP19A was once again determined as a potential regulator of flowering time, supported by the down-regulation of its expression during floral induction of many species (54,57,58). In addition to auxin, flowering time might also be regulated through FCS-like zinc finger (FLZ) light-related proteins. FLZ proteins have been known to interact with genes regulating light-dependent developmental processes (*COL1* and *STH2*) (59). The proteins were also demonstrated to negatively regulate flowering time in rice and *A. thaliana* (60,61). The third candidate gene for flowering time (*ESD4*) had its contribution to the trait's regulation described in *A. thaliana* (62).

Anthocyanins, as a highly abundant group of pigments, confer the distinctive purple color to spinach leaves and petioles, attracting consumers due to both their visual appeal and associated health benefits (63). Previous research shed light on the genetic components underlying anthocyanin biosynthesis in spinach, unveiling a network of 22 biosynthetic genes and 25 transcription factors (TFs) including MYB, bHLH, and WD40 (63). Expanding upon these findings, our XGBoost model for petiole color prediction has provided more insights into the regulatory mechanisms governing spinach pigment production. We identified genes that might affect the signaling pathways of abscisic acid (ABA), a regulator of anthocyanin biosynthesis and accumulation in many species (64–67). The first gene that might affect ABA signaling was *LSM1B*, encoding an Sm-like protein. Proteins in the snRNP Sm family such as *SAD1* have been reported to affect ABA sensitivity and expressions of several genes in the ABA signaling pathway (68,69). The next candidate gene that might be involved in ABA signaling encodes phosphatase 2C, which is inhibited by ABA, releasing SNRK2 phosphorylating ABA-responsive element-binding factors (70–72). In addition to genes related to ABA signaling, four more genes that might be the regulators of spinach anthocyanin biosynthesis and accumulation were found. Particularly, we found the gene encoding the axial regulator YABBY5, which is a positive regulator of anthocyanin and total flavonoid contents in *Artemisia annua* (73). A small HSP encoding gene (*HSP22*) was also found. This might be due to the fact that anthocyanin biosynthesis depends on temperature (74). Other HSPs (like *HSP70*) are also known to affect the anthocyanin level (75). Another candidate gene was *NAC071* encoding a NAC-domain containing protein. Proteins with NAC domain were mentioned as anthocyanin content regulators (76). Finally, we found a candidate gene encoding a serine decarboxylase, the catalyst of ethanolamine biosynthesis (77). Ethanolamine is involved in the biosynthesis of phosphatidylethanolamine which is the precursor of lysophosphatidylethanolamine, the stimulator of anthocyanin accumulation (78–80).

Auxin, cytoskeleton architecture, and cell division have emerged as key players in the regulation of leaf flattening, a phenomenon extensively documented in previous studies (81,82). Our research aligns with these earlier findings, as a substantial proportion of the candidate genes that we identified as putative regulators of leaf surface texture was closely associated with auxin, cell proliferation, and cytoskeleton-related processes. Among these candidates, three genes relating to cell division were found, including *TTM3* (whose 5′ UTR contains an uORF responsible for cell division (83,84)), the gene encoding G2/mitotic-specific-cyclin-1 (a cell cycle controller (85,86)), and a member of the cytochrome P450 gene family (which can affect cell proliferation (87)). Additionally, a cytoskeleton-associated gene was found (*PHOX1*), encoding a protein interacting with myosin, which regulates the straightening of plant organs (88,89). The final candidate gene that might regulate spinach leaf surface texture is *GRXS16*. Glutaredoxins (GRXs) along with thioredoxins (TRXs) control the level of reactive oxygen species (ROS), affecting auxin signaling and cell cycle (90,91).

## Conclusions

Our study presents an answer to the global clustering of spinach, consolidating the observations in previous cluster analyses based on SSR and genomic techniques. Specifically, our findings reveal that cultivated spinach accessions can be classified into three distinct groups, namely Asia, the Middle East, and Europe/US, with the Middle Eastern and European/US accessions exhibiting close genetic relationships. Additionally, our investigation sheds more light on the migration history of spinach by leveraging genomic data, thereby augmenting our understanding of this vegetable's historical migration as documented in historical records. Particularly, our study offers a more comprehensive account of how spinach was introduced to Europe. Finally, our machine-learning-based approach for identifying pivotal genetic variants associated with agronomic traits has successfully generated concise lists of candidate genes that encompass these variants. It exemplifies that using extreme gradient boosting to identify genotype-phenotype relations holds great potential for future studies using similar data sets. Our findings will be of importance for spinach breeding programs aimed at improving traits such as early bolting resistance, petiole color, and leaf surface texture.

## Supplementary Material

lqae034_Supplemental_Files

## Data Availability

The raw data analyzed in this study were generated by Cai *et al.* (6) and are available from the NCBI SRA (BioProject PRJNA598728). Source data files for trees, variant files, and Python code generated for data analysis are available at http://bioinformatics.boku.ac.at/.
