# Supplementary material for: Spinach genomes reveal migration history and candidate genes for important crop traits
Source: NAR Genom Bioinform. 2024 Apr 17;6(2):lqae034. doi: 10.1093/nargab/lqae034 (PMC11023180; doi:10.1093/nargab/lqae034)
Supplement: lqae034_Supplemental_Files [file lqae034_supplemental_files.zip › NguyenHoang_etal_R1_Supplement.pdf]

Supplementary data

Supplementary Figures

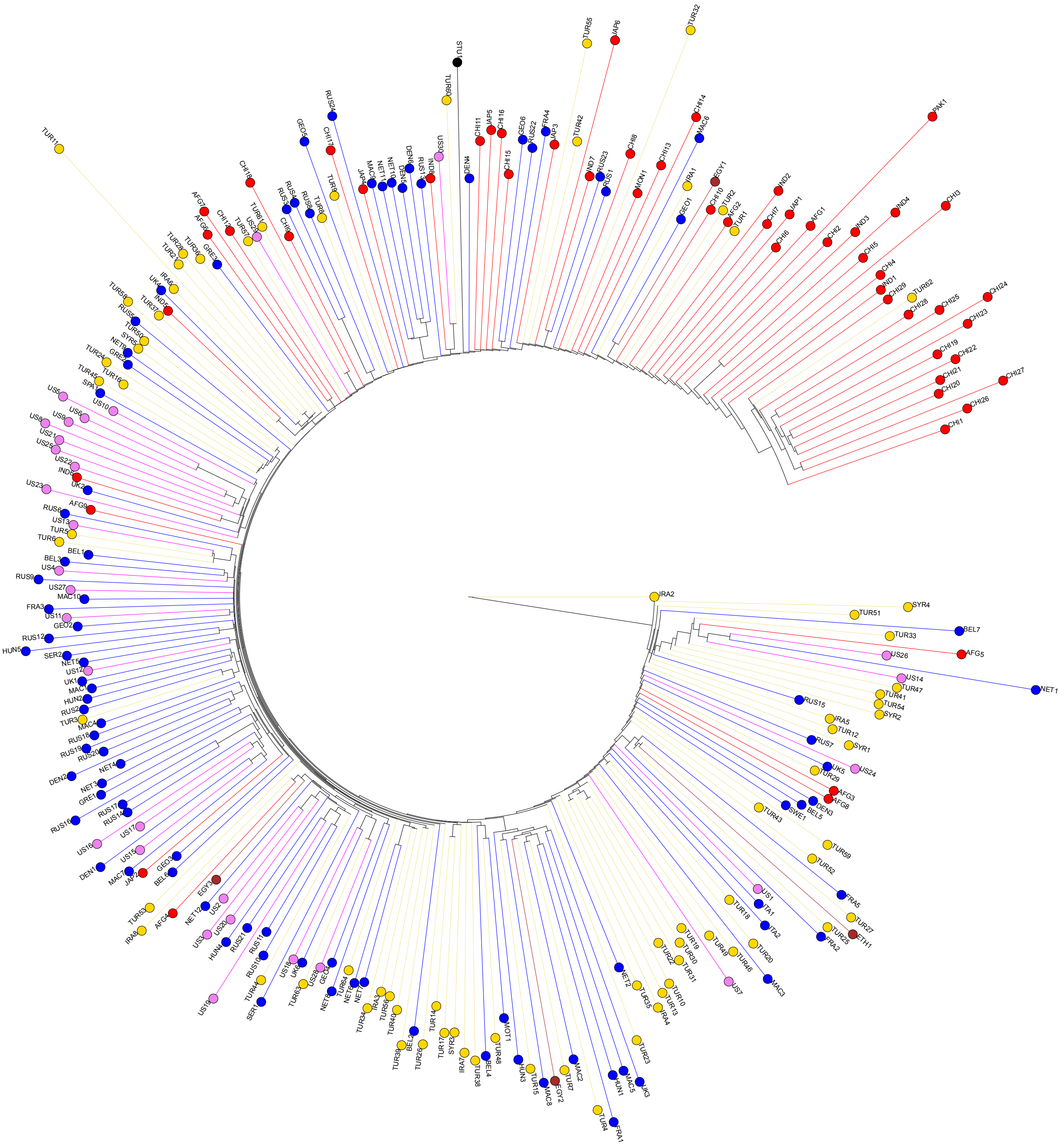

Supplementary Figure 1: K-mer-based tree rooted at IRA2 showing 256 cultivated spinach accessions from Asia (red), Europe (blue), the Middle East (yellow), the US (pink), Africa (purple), and one wild accession (STU1).

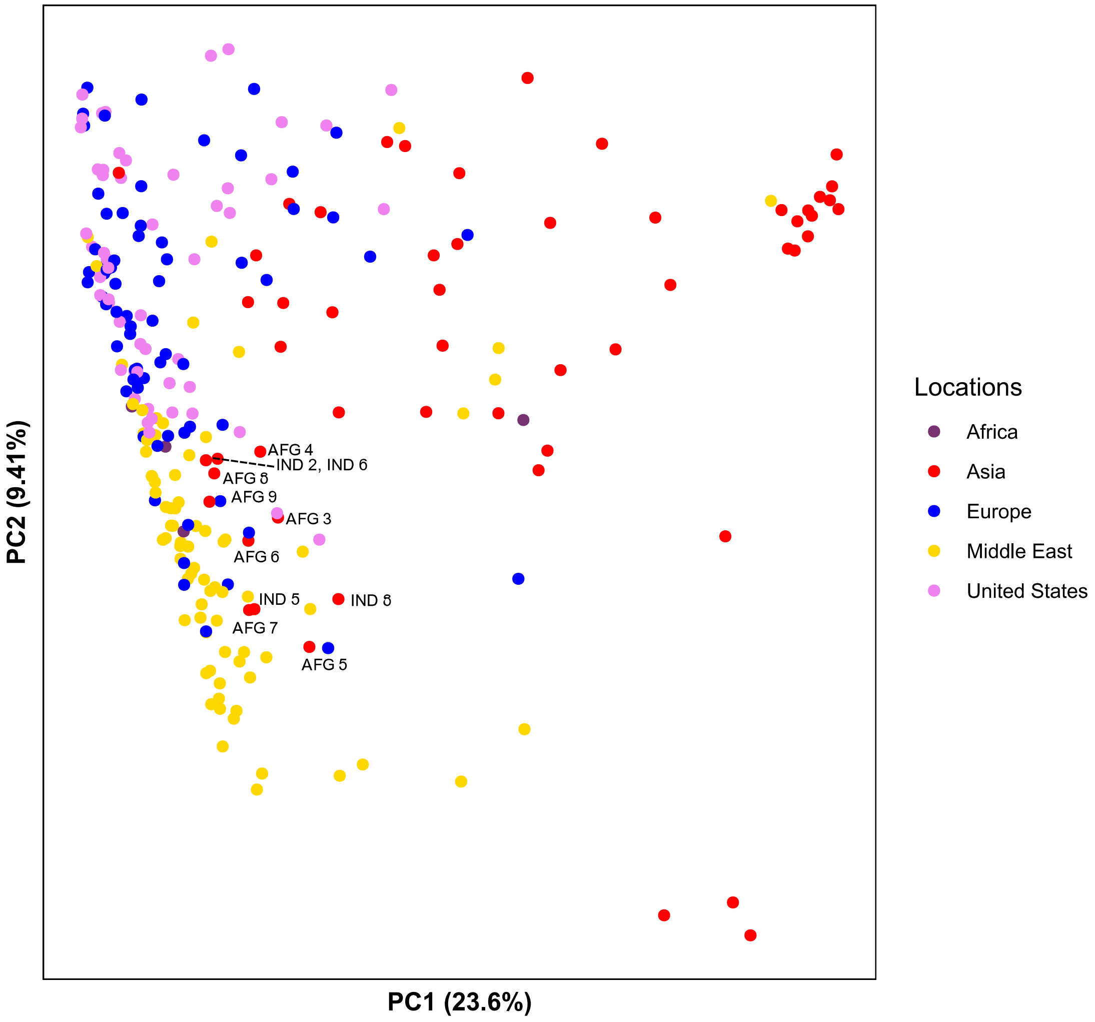

Supplementary Figure 2: PCA plot with labelled accessions from Afghanistan (red dots) that were close to the Middle Eastern cluster (yellow dots).

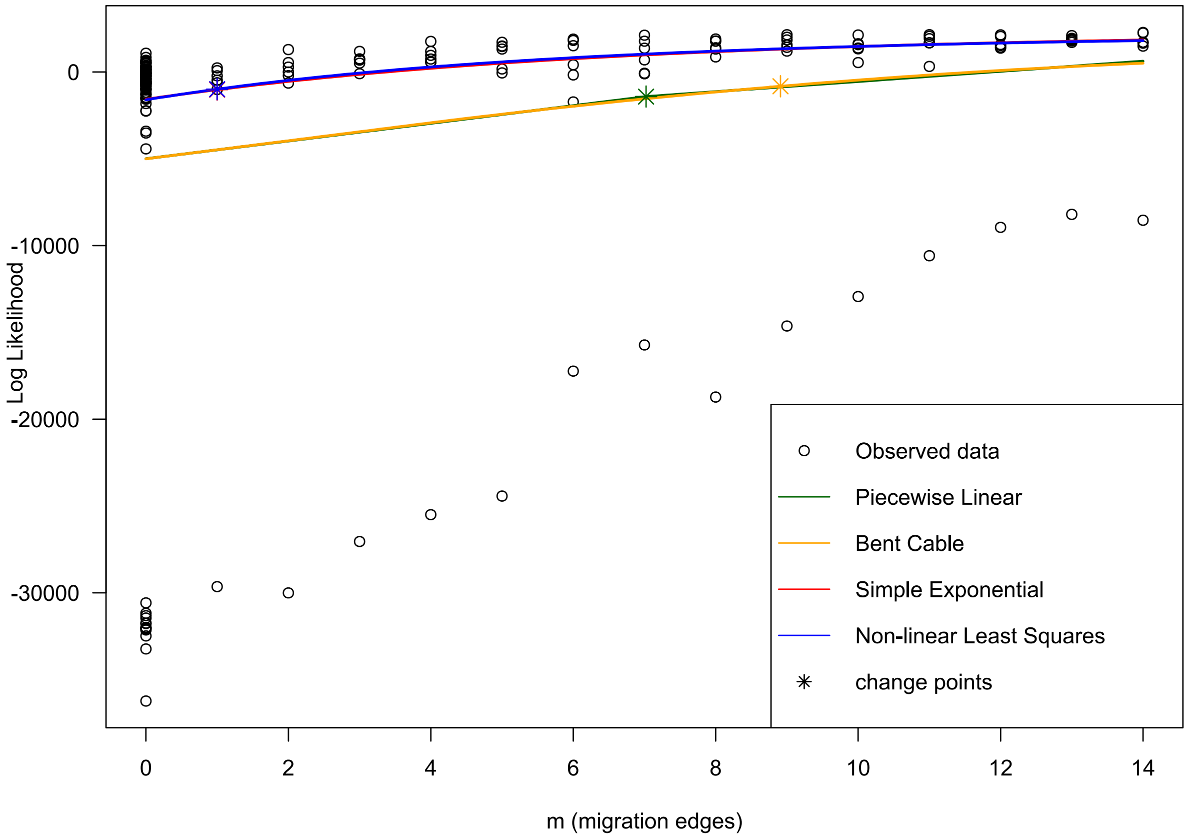

Supplementary Figure 3: Visualization of four different models fitted to the likelihood scores of 98 Treemix runs. The best fit model was the Simple Exponential model and the Non-linear Least Squares model, with the optimal number migration edges of 1.

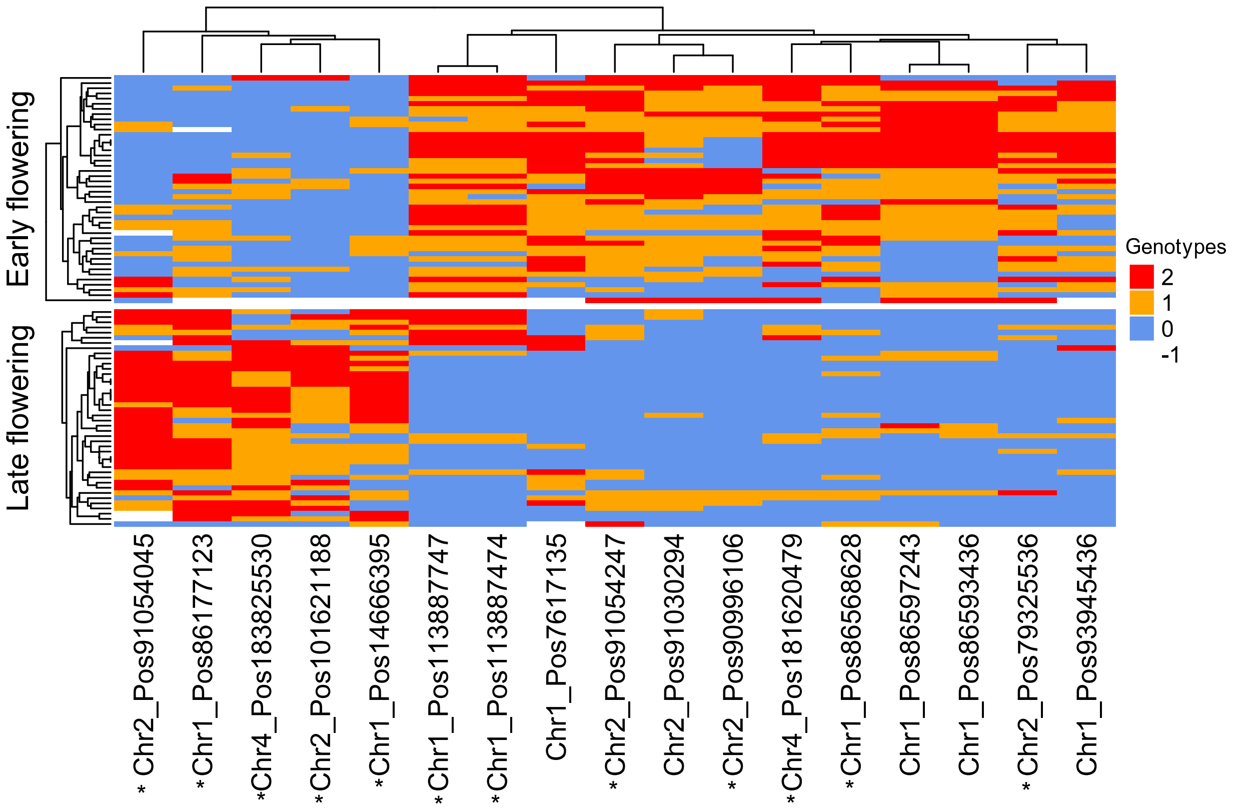

Supplementary Figure 4: Genotype distribution patterns among phenotypic groups associated with flowering time. The genotypes were recoded as “0” for homozygous reference, “1” for heterozygous, “2” for homozygous alternative, and “-1” for missing genotype. The horizontal axis represents variant positions, and the vertical axis shows the individuals in each phenotypic group. Variants associated with genes are highlighted with asterisks.

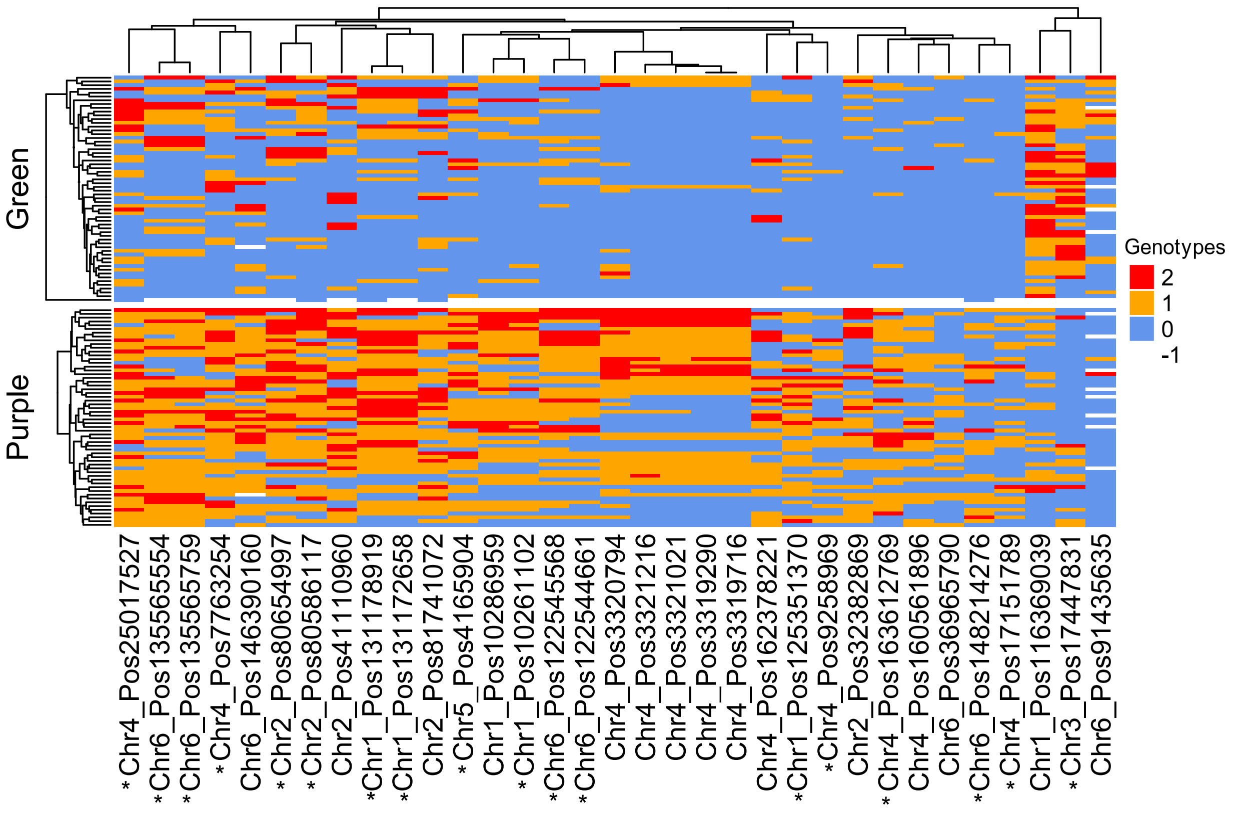

Supplementary Figure 5: Genotype distribution patterns among phenotypic groups associated with petiole color. Details as in Supplementary Figure 4.

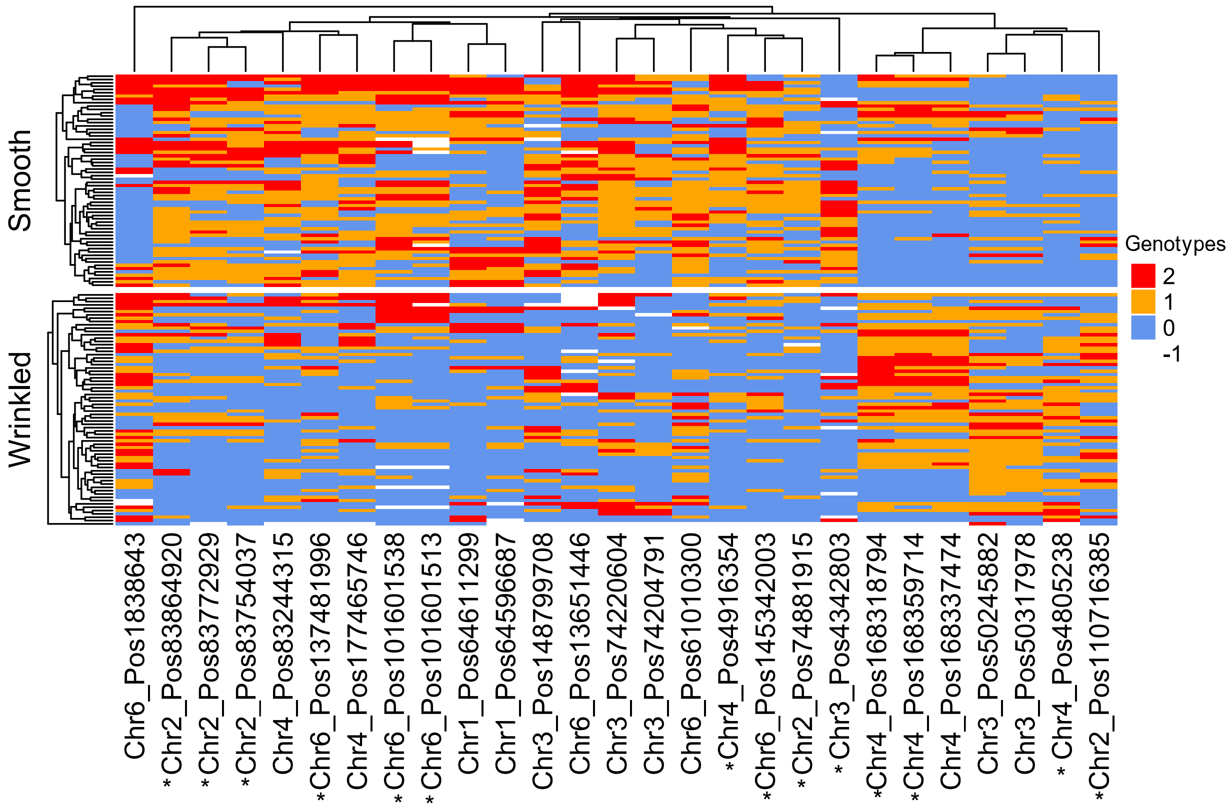

Supplementary Figure 6: Genotype distribution patterns among phenotypic groups associated with leaf surface texture. Details as in Supplementary Figure 4.

Supplementary Tables

Supplementary Table 1: Details on spinach accessions analyzed in this study.

Supplementary Table 2: Coordinates of data points in the PCA plots.

Supplementary Table 3: Fitting different models to 98 Treemix runs. The optimal number of migration edges are the points where no further significant increment in the likelihood score was detected. The best-chosen migration edge number associated with the model that best fit the Treemix outputs (with the lowest AIC). AIC, Delta AIC, and df represent the Akaike information criterion, the difference between a specific model's AIC value and the smallest AIC value, and the degrees of freedom, respectively.

Supplementary Table 4: Calculated F4 statistics for migration path validations.

Supplementary Table 5: Variant positions deemed crucial for phenotypic data predictions using the XGBoost models and additional correlated variants. Variants were assigned to a gene including the region of 5 kbp up- and downstream. A variant was assigned to each gene if there was more than one gene in such a region.
